# Factors Influencing Consumer Behavior toward Green Products: A Systematic Literature Review

**DOI:** 10.3390/ijerph192416568

**Published:** 2022-12-09

**Authors:** Andreea Barbu, Ștefan-Alexandru Catană, Dana Corina Deselnicu, Lucian-Ionel Cioca, Alexandra Ioanid

**Affiliations:** 1 Faculty of Entrepreneurship, Business Engineering and Management, University Politehnica of Bucharest, RO-060042 Bucharest, Romania; 2 Academy of Romanian Scientists, RO-050044 Bucharest, Romania; 3 Faculty of Business and Administration, Department of Business Administration, University of Bucharest, RO-030167 Bucharest, Romania; 4 Faculty of Engineering, Industrial Engineering and Management Department, “Lucian Blaga” University of Sibiu, RO-550025 Sibiu, Romania

**Keywords:** green products, factors, behavior, consumer behavior, systematic literature review

## Abstract

In the last few decades, humans have consumed more resources than in all of previous history. Hence, we are living in times in which the topic of environmental protection is a global concern. The paper aims to conduct a systematic literature review on consumer behavior, as well as identifying the main factors that interfere with consumer behavior toward green products. A total of 37 studies were found and systematized using inclusion and exclusion criteria. The papers were selected only if they featured research on consumer perceptions of green products. Using this search strategy, a literature analysis was performed based on papers extracted from Web of Science, Emerald Insights, Springer Link, and Science Direct. As a result, various factors that influence consumer behavior toward green products were identified, such as social norms, natural environmental orientation, the company’s perceived green image, green product characteristics, perceived risks and inconvenience of buying green products, perceived benefits of buying green products, institutional trust, sociodemographic characteristics, and consumer confidence. Even though completing a systematic literature review is not something new in academic research, the novelty of this paper is found in its theme: consumer behavior toward green products. Although the analyzed articles highlight quite varied reasons, the articles emphasize that the green products should take into account the needs, expectations, and perceptions of customers.

## 1. Introduction

In the last few decades, humans have consumed more resources than in all of previous history. Hence, we are living in times in which the topic of environmental protection is a global concern. There are estimations that almost 70% of the planet’s greenhouse gas footprint depends on which products customers choose and whether they use and dispose of them in a sustainable manner [1]. Thus, consumers are becoming more interested in environmental topics and reflecting their interest in their decision to buy green products [2]. The United Nations Environment Programme (2011) [3] has estimated that the green products market doubles annually. Another international study has indicated that 73% of consumers across 60 countries are determined to pay more for sustainable products [4]. In a survey developed by Harvard Business Review [1], 65% of the consumers surveyed said they want to buy eco-friendly products, but only 26% of them do so, thus this is a contradictory desire.

The topic of increasing consumer interest in green products has rapidly expanded globally over the last few years [5]. The global market for green products is estimated at USD 44 trillion, more than half of global GDP [6]. The global market for low-carbon environmental goods and services is estimated at EUR 4.2 trillion [7]. Moreover, the global green technology and sustainability market size was valued at USD 11.49 billion in 2021 and is projected to grow to USD 13.76 billion in 2022 [8]. This phenomenon is facilitated by the COVID-19 pandemic when the heightened attention to hygiene and wellness led to increasing demand for safe products for both consumers’ families and the environment [9]. In Romania, a European Union country with an emerging economy, 73% of the consumers state that they are willing to pay more for green food grown without chemical fertilizers and pesticides, 49% would agree to pay more for renewable energy, and 36% would pay more for products with biodegradable components [10].

The state-of-art review confirms the absence of a universal, effective, and well-structured definition of a green product [11,12,13,14]. Although there is no internationally recognized definition, a green product (or ecological product/eco-friendly product/sustainable product) is “a sustainable product designed to minimize its environmental impacts during its whole life-cycle and even after it is of no use” [15]. In essence, a green product has two main characteristics: reducing waste and maximizing resource efficiency [16]. The literature analyzed focused on green products from a variety of viewpoints, presenting both their benefits and barriers [17]. Researchers have linked ecological products with environmental protection [18], sustainability [19], reduced wastage during production [20], social quality [21], economic benefits [22], low energy consumption [23], low emissions [24], less packaging [14], etc. However, other authors have revealed the negative aspects of sustainable products as follows: higher price [25], costly green certifications [26], and the short durability of the product [27].

Many researchers have explored consumer behavior toward the green products topic and have presented different viewpoints on this subject. First of all, the green products consumers use contribute to the protection of the environment by consumers’ refusal to buy products that are harmful to the environment [28,29]. Secondly, a green consumer can be defined as an individual who adopts environmentally-friendly behaviors and buys green products rather than standard products [30]. Thirdly, consumers with environmental awareness are not only interested in the consumption process, but also in the production, the scarcity of consumed resources, and post-use processes of products [31,32,33,34,35,36,37,38].

Against this background, this paper seeks to identify and analyze the factors that influence consumer behavior toward green products. To achieve the aims of the paper, the authors employed a systematic literature review. Even though completing a systematic literature review is not something new in academic research, the novelty of this paper is in its theme: consumer behavior toward green products. Although the analyzed articles highlight quite varied reasons, the topic of green products should take into account the needs, expectations, and perceptions of customers. Moreover, to determine the main factors that influence consumer behavior toward green products, the authors of this paper also analyzed if the factors treated by each of the analyzed paper confirmed the existing correlations between these factors and the consumer behavior toward green products.

This paper is structured as follows. Section 2 presents the Materials and Methods. The Results and Discussion are presented in Section 3 and Section 4, respectively. Section 5 summarizes this study and illustrates the Conclusions, along with their limitations and research perspectives.

## 2. Materials and Methods

**Figure 1:**
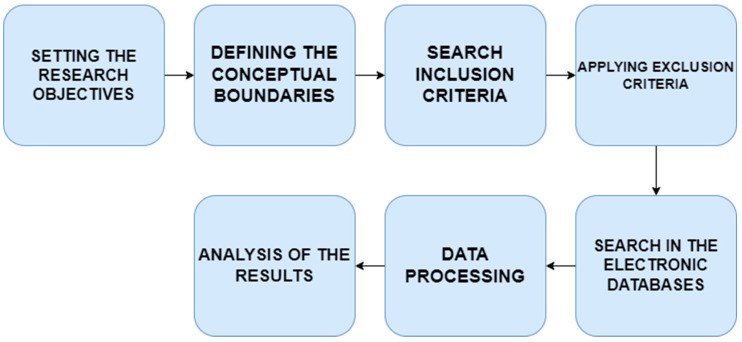
Phases of the systematic literature review.

In order to achieve the aims of the paper, the authors carried out a systematic literature review that encompassed several phases (Figure 1), based on the methodology presented by Glogovețan et al. (2022) [34]. Firstly, the authors of this paper designed the plan for the scientific research by setting the research objectives. Secondly, they defined the conceptual boundaries. The main identified research directions are composed of consumer behavior regarding green products.

**Figure 2:**
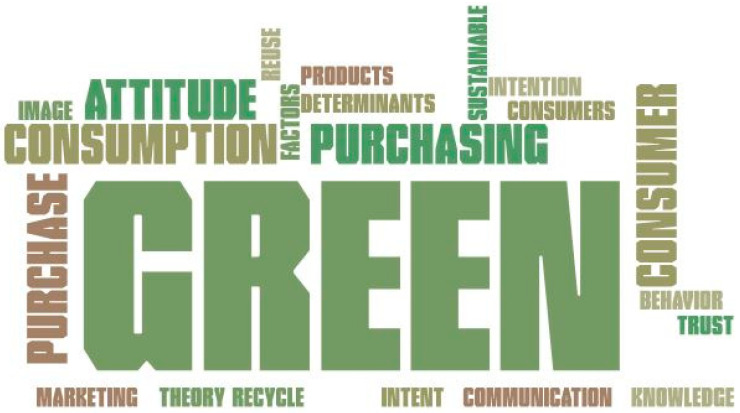
Keywords used for search strategy.

Thirdly, the authors used the inclusion and exclusion criteria reported by [34,35] and they performed a literature analysis through a combination of the following keywords (Figure 2) in several electronic databases such as Web of Science, Science Direct, Springer Link, SAGE, and Emerald Insight.

The initial search generated 119 papers, of which 37 titles fit the considered criteria (Table 1); therefore, they were analyzed further. The articles were evaluated to identify if they deal with consumer perception on green products.

The publications were selected only if they featured research on consumer perceptions regarding green products and were retained for further analysis only if they simultaneously fulfilled the eight methodological criteria (Table 1) proposed by [34] and [35]. The final set of articles included in the present systematic literature review consists of 37 publications.

## 3. Results

To achieve the goal of this paper, the authors divided the results of their analysis into three sections. The first section presents the methodology used in the reviewed studies and other significant information about the analyzed articles. The second section presents the main factors influencing consumer behavior toward green products observed by the authors in the reviewed studies, while the third part contains a brief analysis of each determined dimension.

### 3.1. Revision of the Studies

Out of the 37 reviewed articles (Table 2), 5 of them were published in 2022, and 13 were published in 2021, while the rest covered the years 2017–2020. These articles were published in journals with the main focus being on business strategy and the environment (14), sustainability (11), consumer services (5), cleaner production (4), environmental psychology, and public health (3). The majority of the papers presented quantitative studies, with some of them also containing qualitative research.

Out of the 37 papers, 4 of them presented a literature review about different variables that can influence green product purchasing behavior. Moreover, in these papers, a variety of statistical procedures were used, such as the ANOVA Kruskal–Wallis test, the Mann–Whitney U test, Pearson’s Chi-square test, Exploratory Factor Analysis, Kaiser–Meyer–Olkin test, personal interview and focus group discussions, multivariate data analysis techniques using structural equation modelling, and multiple regression analyses.

### 3.2. The Main Factors Influencing Consumer Behavior toward Green Products

**Figure 3:**
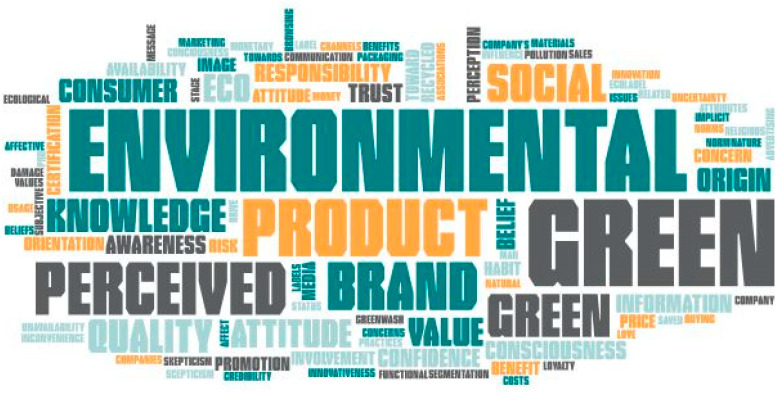
Factors influencing consumer behavior toward green products.

The analysis of the reviewed papers revealed a series of factors that can influence consumer behavior toward green products. 111 factors were analyzed, with most of them being quite similar in form and meaning. However, the authors of this paper were able to group these factors according to their form and meaning into eight main categories: social norms, natural environmental orientation, a company’s perceived green image, green product characteristics, perceived risks and inconvenience of buying green products, perceived benefits of buying green products, institutional trust, sociodemographic characteristics, and consumer confidence (Figure 3):

Regarding the results of the analyzed studies, the authors of this paper also analyzed if the factors assessed by each paper confirmed the existing correlations and the buying behavior toward green products. Comments based on these correlations will be made in the following subsections, for each main category of identified factors.

### 3.3. Social Norms and Consumer Behavior toward Green Products

The reviewed papers highlighted that in the context of green product consumption, social norms describe the way society views environmental issues [47]. In addition, social norms refer to how people think and act pro-environmentally [72]. The reviewed studies present the role of social norms in predicting pro-environmental behavior and changing people’s green behaviors [41,47,53,62,72]. The results indicate that people are significantly influenced by society’s actions regarding pro-environmental issues and the way society presents normality in this context.

The factors that were grouped in this category also refer to green product knowledge [40,50,63,65], natural environmental orientation [67], green habit [67], knowledge about environmental damage and pollution [71], attitudes toward environmental protection [71], environmental concern habit [40], environmental consciousness [37,58,61,64], attitudes toward environmental issues [64], green trust [40,50,54,66] green involvement [54], environmental beliefs [59,60], environmental awareness [5,50,59], environmental concerns [36,58,59], green attitudes [50,61], drive for environmental responsibility [36,42], perceived ecological value [44], green practices [46], green product awareness [44,45], and consumer social responsibility [54].

All of the identified factors influence consumer behavior toward green products. Thus, to make positive changes in people’s behavior toward green products, it is necessary to make changes at a societal level regarding the attitudes toward environmental issues and also to educate people in this regard.

### 3.4. A Company’s Perceived Image and Consumer Behavior toward Green Products

Corporate image refers to the overall impressions of the organization’s stakeholders, as well as how stakeholders perceive the firm as a business [38].

In the last few years, environmental issues such as global warming and environmental damage have represented real concerns for people all over the world [73]. People have tried to be more responsible with their actions and have attempted to buy environmentally-friendly products [74]. In this context, companies have been forced to modify their services or products, offering clients solutions that would also meet the environmental protection needs. Thus, they have started to invest in building a strong green image or a green brand for their businesses, and therefore they are trying to influence the consumer behavior of buying green products [75].

The reviewed papers that addressed this topic also highlighted that a company’s perceived green image is built around several concepts such as eco-certification origin [43], brand image [40,48], brand love [48], brand loyalty [48], brand trust [66], green brand associations [5], green brand attitude [5], green brand knowledge [66], green communication [39], social media [42], and product innovation and segmentation [50].

However, not all the studies demonstrated that all of these concepts influence consumer behavior toward green products. For example, [40], as well as [69], argues that green brand knowledge, environmental knowledge, consumers’ environmental attitudes and green knowledge do not influence attitudes toward using green products, but investing in brand trust and brand image are key actions that influence consumer behavior [5].

### 3.5. Green Product Characteristics and Consumer Behavior toward Green Products

It is almost impossible to talk about green products without mentioning their characteristics or attributes. Cheung and To (2019) [64] found out that green product information can be a key determinant of consumers’ green purchase behavior. Zhang and Dong (2020) [55] claim that a green product needs to be credible in order to be appreciated by potential buyers, so the eco-label can affect the consumers’ buying behavior [55]. In addition, besides eco-labels, consumers are taking into account other aspects such as the quality of the green products [45,51,55,57,70] or the awareness about green products [44,45,54,55], with all of them having a significant impact on consumer behavior toward green products.

After analyzing the selected papers, the authors of this research concluded that consumer behavior toward green products can be influenced by the following product characteristics: price [45,57], quality [45,51,54,55,56,57,64,70,71], availability [45,46,55,68], packaging [55], eco-label [45,46,55], material [46], functional value [46], and green product information [64].

The availability of green products is an important factor that can positively influence consumer behavior toward green products [45]. This factor can also influence the consumers’ buying behavior, because they can choose between different types of green products or different brand names. In addition, the perceived quality of green products can impact consumers’ green purchasing intentions [46,64], with the packaging also playing an important role in influencing the perceived quality of green products [45].

### 3.6. Perceived Risks and Inconvenience of Buying Green Products and Consumer Behavior toward Green Products

Perceived risk refers to the subjective evaluation of customers regarding the possible consequences of wrong purchasing decisions [70], while perceived inconvenience of buying green products refers to aspects such as: the price, difficulty in evaluating them depending on how ecological they are, difficulty in finding green products in regular stores, and additional time and effort to get to the specialized stores where they are marketed [60].

In their research, Caniëls et al. (2021) [60] started from the idea that higher perceived inconvenience is associated with a negative attitude towards buying green products. Their paper highlights that green products are more expensive than normal products, they are not easy to find in stores and it is also hard to determine their actual degree of “greenness”. Therefore, consumers need to make an extra effort in order to find green products, to analyze the specifications written on the label and their relevance, and of course, to buy them at a higher price. Furthermore, the analyzed paper also focuses on the relationship between the inconvenience of buying green products, pro-environmental beliefs, and social values. According to the consumers’ pro-environmental beliefs, the green buying behavior of young people who perceive the high inconvenience of buying green products is largely influenced by the social value attached to buying green products [60]. Moreover, Wasaya et al. (2021) [51] state that those customers who attach great importance to the environment, who are aware of its problems, have a risk perception in their mind that the product or service they use will not be according to the claims they have made. This situation generates uncertainty and brings negative changes regarding the attitudes of customers regarding the purchase of products or services. Thus, the perceived risk becomes a subjective assessment of potential clients that is associated with the possible consequences of wrong decisions. Regarding the types of perceived risks, Wang (2017) [70] states that they can refer to product performance, social aspects, psychological aspects, physical aspects, and loss of time.

Regarding this section, by analyzing the selected papers of this study, the authors found out that consumer behavior toward green products can be influenced by the following factors: green perceived risk [51,70], perception of recycled product risk and uncertainty [71], and perceived inconvenience of buying green products [60].

Sun et al. (2018) [71] analyzed the case of recycled products and discovered that the perception of recycled products risk is negatively related to the perception of quality for recycled products and positively to the pro attitude toward environmental protection. Their results showed that concepts such as risk and uncertainty associated with green products negatively influence consumers’ buying behavior. Wasaya et al. (2021) [51] also highlight that there is a negative and significant relationship between environmental awareness and green perceived risk.

### 3.7. Perceived Benefits of Buying Green and Consumer Behavior toward Green Products

Regarding the attitude toward eco-social benefits, Cheung and To (2019) [64] claim that this concept measures the degree to which the potential consumers of green products agree that purchasing those products will bring social benefits and have a positive moral value.

When it comes to the relationship between the benefits of green products and consumer behavior toward green products, the authors of this paper extracted from the selected papers some important factors that can be grouped into a single dimension: attitude toward eco-social benefits [64], company benefit belief, personal benefit belief [47], and perceived consumer effectiveness [38,40]

Concern for the environment may lead consumers to maximize the eco-social benefits. Buying eco-friendly products brings them more psychological benefits, and they perceive life improvement through using green products [64]. In addition, as green products consume less energy, consumers believe that green products can bring even more benefits, while their attitude towards environmental protection will also be improved. Therefore, this study asks how a consumer’s target knowledge (the belief that the consumer will benefit from good quality) and agent knowledge (the belief that the company will benefit from it) are associated with their purchase intentions from companies, following environmentally sound practices.

Besides personal benefit beliefs, company benefit beliefs significantly predicted purchase intentions [47]. In their research, Ham et al. (2022) claim that the company benefit belief is one of the strongest predictors of purchase intentions across all generations. In the case of the companies that use corporate social responsibility and also green marketing, potential customers are more attracted to buy from them and seem to appreciate their efforts to be eco-friendly. Moreover, if a customer believes that a company implements green practices, he/she is willing to support that company and buy green products from it [47].

Perceived consumer effectiveness becomes an important predictor of green purchase behavior, with it being even more significant than environmental attitudes alone [38]. Perceived consumer effectiveness positively influences green purchase behavior and also the company’s perceived green image [38]. Wang et al. (2019) [40] highlight the fact that companies should attach environmental protection labels to green product packaging to enhance perceived consumer effectiveness. Thus, by specifying such information on the label, such as resources saved or carbon emissions reduced, companies can encourage consumers to contribute to the protection of the environment by purchasing green products.

### 3.8. Institutional Trust and Consumer Behavior toward Green Products

Institutional trust refers to trust in institutions, whether we are considering governments or companies. This is an important aspect to be studied in the case of green product buying behavior, mainly when consumers do not have enough knowledge or time to analyze their options regarding green products [68].

In the papers selected for this study, the authors found that there is a significant relationship between institutional trust and consumer behavior toward green products, with this relationship being mediated by factors such as institutional trust [68] and government support [44].

In their paper, Ricci et al. (2018) studied the trust-to-go-green concept and discovered that institutional trust in food supply chain-related actors influences the attitudes of consumers towards green products and also the concern about the environmental and health-related impacts of agricultural practices. Their results indicate that the more consumers trust supply chain-related actors, the more they would be willing to buy green food [68].

An important role is played by government support, which should act in the sense of promoting a sustainable market, and positively influencing consumer attitudes toward green products [76]. Al-Kumaim et al. (2021) [44] analyzed the relationship between government support and purchase intention toward green products purchase behavior. According to them, one of the government’s roles is to provide guiding principles to consumers that can affect their buying attitudes based on the health and safety values of green products. In addition, the government’s policies regarding the environment positively impact consumers’ attitudes toward green products [44,77] the government’s support and institutional trust play an important role in consumer behavior toward green products.

When green companies manage to raise the consumers’ level of trust in institutions, they can also improve the consumers’ probability to choose green products [68]. Thus, institutional trust can be considered as an important factor that can be used to promote green acquisitions to consumers who are not particularly involved in eco-friendly behavior [68].

### 3.9. Sociodemographic Characteristics and Consumer Behavior toward Green Products

Sociodemographic characteristic seems to also be an important factor that can influence consumer behavior regarding green product acquisitions [52,55]. This dimension was represented in the specialty literature by items such as age [47], gender, education, number of children [40], place of residence, or financial situation [40,52].

When it comes to the influence of gender on green product acquisition, Witek and Kuźniar (2021) [52] highlighted the fact that women are more sensitive about environmental issues than men, more interested in health and living in a safe environment, and more interested in buying eco-products than men, even if they need to pay higher prices for green products.

Even if some pieces of research have demonstrated that age does not influence green behaviors [36], the results obtained by Witek and Kuźniar (2021) [52] show that the older age the of consumers, the greater the social pressure for purchasing green products, and older consumers expressing green purchase intentions [47]. Thus, older people seem to be more responsible in addressing environmental problems through actions than the younger generation [47].

Regarding the impact of education on green product acquisition, there is still room for improvement. The results obtained by Witek and Kuźniar (2021) [52] did not support the hypothesis according to which education has a positive impact on the consumers’ green purchase intentions, while other studies show the opposite [40,78,79].

Income [40] and personal financial situation are factors that can influence green behavior too, as the personal financial situation highly influences the consumers’ willingness to pay more for green products [52]. The number of children has an impact on the frequency of green product purchases as well, as mothers are very concerned about their children’s health and safety, which is why they tend to buy more green products [52].

### 3.10. Consumer Confidence and Consumer Behavior toward Green Products

Consumer self-confidence is associated with buying intention, as well as green product quality and price perceptions [57]. Self-confidence is related to a positive experience in supermarkets or other marketplaces [76].

By analyzing the papers selected for this research, the authors of this paper discovered that some similar concepts that can influence consumer behavior toward green products could be grouped into consumer confidence [37], consumer protection confidence, and consumer self-confidence [57].

To predict consumers’ purchase decisions, it is necessary to understand consumer confidence, which is an indicator of customers’ evaluations of the national economy and their financial situation [80]. In their research, Han et al. (2022) [37] discussed consumer confidence and its positive relationship with green purchase intention. They found that this relationship is partially mediated by environmental and status consciousness. Moreover, they highlighted that the results of their paper can help retailers in developing better marketing tactics by understanding how consumer confidence fluctuations can influence the major economic growth-driven environmental challenges.

In their paper, D’Souza et al. (2020) [57] discussed the level of the consumers’ perceived ability to make sound decisions and also the ability to acquire and use information by looking at consumer self-confidence and consumer protection self-confidence.

When it comes to consumer self-confidence, manufacturers, and retailers need to analyze the consumers’ confidence in their ability to obtain and understand green product information, their ability to choose the right product based on that information, their confidence regarding how their choices correspond with their expectations, and how they can improve satisfaction [57].

Similarly, regarding consumer protection self-confidence, it is important to analyze the marketers’ strategies used to persuade consumers to buy green products and also the level of consumers’ confidence in dealing with these strategies and consumers’ rights. Thus, through their paper, D’Souza et al. (2020) [57] claim that consumer self-confidence moderates the relationship between the price and the quality of green products in the decision to buy green products, highlighting the fact that consumer protection confidence has no moderating effect on these relationships. In addition, consumer self-confidence can modify consumers’ purchasing intentions.

## 4. Discussion

This systematic review reveals the most important factors influencing consumer behavior of green products. From the analysis of the selected 37 papers, a series of common characteristics were identified, highlighting various representative factors for this field, even if the methodologies used in these studies were different or the results obtained were somewhat contradictory regarding the relationship between the studied variables. Thus, the most used factors in influencing user behavior toward green products could be grouped into eight distinct categories: social norms, a company’s perceived green image, green product characteristics, perceived risks and inconvenience of buying green products, perceived benefits of buying green products, institutional trust, sociodemographic characteristics, and consumer confidence.

This grouping made by the authors of this paper, as well as the establishment of the eight categories of factors, can be seen as a new contribution to the specialty literature, thus coming in as a gap analysis that has the role of both filtering, extracting, and presenting the most representative factors that influence consumer behavior regarding the purchase of green products.

Although in the past, social norms represented one of the most used factors influencing consumers’ environmental behaviors [81], this concept is still an important one when it comes to making consumers act pro-environmentally [41,47,53,62,72,82]. Letting people know how others act regarding the protection of the environment can be the key to how social norms can influence green buying behavior [81]. Consumers need to choose products that do not harm the environment and take care of their buying behavior as well. Their attitude towards using and buying green products can influence other members of society, which is why they need to be responsible for their actions. Consumer social responsibility is positively related to green purchasing intention [54], therefore “people must be aware of their role in reducing ecological issues and respond appropriately” [83].

Even if the majority of the papers from our study have shown that the subjective norms directly affect consumer behavior toward green products [36,51,54,61,64,65,66,67,71], there is evidence to support the fact that this factor does not directly impact green purchase intention, but it indirectly relates to future purchase intention [38,43,66]. This relationship is mediated by a company’s perceived green image when a green corporate image is a long-term goal for the management. In a competitive market, the green image could play a key role in building a company’s brand, which can differentiate a company’s offerings from competing ones [47], with the image of a company being valuable for increasing customer satisfaction [84]. Thus, companies should focus on communication and green marketing techniques which deliver information that influences consumer behavior to be more eco-friendly [85].

Also, making consumers aware of green product information can be a key determinant of green purchase behavior [64]. Most of the time, when customers are concerned about the environment and its preservation, they tend to buy green products if these products have eco-certifications or are produced under eco-certification schemes and respect rigorous regulations [45,46,49,55].

For the potential customers who find the green product characteristics on the eco-labels, the decision to buy the product is easy to make, with this analysis making them pay even higher prices for green products that might cause less harm to the environment [64]. Thus, if the information regarding green product characteristics convinces consumers that their attributes, designs, and functions are beneficial to them or the environment [45], people are willing to pay more for that functional value.

When it comes to green product buying behavior, perceived risk is also a factor that should be analyzed [51,60,70,71]. This perceived risk is consumers’ valuation, which is associated with the possible consequences of wrong decisions [86]. The analyzed scientific papers of this research topic highlight that risks and uncertainty regarding green products negatively influence potential customers’ buying behavior, with environmental awareness being an important factor that can affect green perceived risk [51]. Therefore, in order to reduce risks, consumers can use different strategies, such as pre-purchase deliberation, seeking additional information, reliance on brand image, personal recommendations, or the security of warranties [70].

Also, the perceived benefits of buying green products can influence consumers’ behavior toward green products [38,40,47,64]. Perceived consumer effectiveness refers to the consumer’s belief that the efforts of his/her actions can make significant differences in solving environmental problems [87]. Therefore, consumers who think that their choices to buy green products will bring benefits to the environment tend to buy more green products [40].

Although trust is considered to be an important factor that can influence consumer behavior [88,89], there is still a lot of debate about how institutional trust can affect consumer behavior toward green products, especially when trust levels may be influenced both positively and negatively [90]. Ricci et al. (2018) [68] highlight the fact that institutional trust can influence consumer risk perception and attitudes, with the level of trust negatively affecting those variables [91]. Concerning the relationship between trust and consumer attitudes, several studies have shown that the higher the level of consumer trust, the higher the probability to develop a positive attitude and, thus, choosing products with eco-friendly characteristics [92,93,94,95].

This research also revealed that sociodemographic characteristics can be important factors that influence consumer behavior toward green products [52,55]. While the results obtained by Witek and Kuźniar (2021) [52] showed no relationship between the consumers’ education and their buying behavior toward green products, other studies claim the opposite [58,78,79]. In addition, sociodemographic characteristics such as gender [47], age [52] or personal financial situation [47] can influence consumer behavior toward green products, while other studies claim they are not important when it comes to the final decision of buying green products [63,96,97]. Moreover, our analysis showed that there is a positive relationship between consumer confidence and consumer behavior toward green products [37,57], with this being one of the factors that can significantly influence potential customers’ behavior.

The papers from the specialty literature that separately analyze the interaction between various factors and the attitude of consumers and their behavior regarding the purchase of green products make the mission of researchers, and also merchants, quite difficult in terms of understanding and managing these situations. On one hand, researchers who want to make important contributions to the green products industry should start from a clear set of factors that generally influence the purchase of green products. After understanding these concepts and how they are correlated with the perceptions of potential consumers, they should customize them for specific green product industries and test if the same types of correlations exist in their case. However, if there is not a clear enough basis regarding the main factors from which their analysis must start, then the future results that will be obtained by them could be incomplete or less relevant for all those who want to know more about generic green products. On the other hand, green product traders must research the existing case studies in the specialized literature to analyze the type of variables taken into account for testing the conceptual models presented in the scientific works. They must understand the basic concepts used, check if they all match their types of specialized green products, and if the conclusions and directions formulated by the authors of the papers present information that could be useful to them in the case of the industries in which they are used. The efforts of retailers should first focus on papers that present the basic concepts absolutely necessary in managing the behavior of customers interested in green products, and only then should they focus on specific case studies. Thus, this paper comes as a cornerstone in the green products industry in order to be able to create an overview necessary for both researchers and green product traders in relation to the main factors that can determine the purchase of green products.

## 5. Conclusions

Considering that in the last few decades humans have consumed more resources than in all of previous history, the topic of environmental protection is a global concern. In this vein, consumers’ interest in green products has witnessed an impressive rise.

From a theoretical point of view, this paper provides valuable new insights into the expansion of the scientific literature on consumer perceptions on green products. In this line, the green products acquisition process should take into account the factors influencing consumer behavior. Moreover, the paper discusses the positive and negative influence of several key factors that influence consumers’ behavior toward green products: social norms, natural environmental orientation, company’s perceived green image, green product characteristics, perceived risks and inconvenience of buying green products, perceived benefits of buying green products, institutional trust, sociodemographic characteristics, and consumer confidence.

From a practical point of view, the topic of green products should take into account the needs, expectations, and perceptions of consumers. Firstly, by taking into consideration all the factors influencing consumer behavior, companies from the ecological sector should design and implement several strategies to target interested consumers. Secondly, government authorities should support and promote a culture of green products. Thirdly, companies should create a good institutional image, highlighting that consumers should choose products that do not harm the environment.

The present study has certain limitations. First of all, considering the research strategy (which takes into account only papers that fit into the mentioned criteria), the authors of this paper may have omitted some pertinent articles. This brings about the possibility that the removed papers include information that could affect our conclusions. Secondly, there is a need for future research related to factors influencing the consumer behavior toward green products, considering the discrepancy between nations or areas. Thirdly, since this study was based on only some of the factors that influence consumer behavior toward green products, future research should be conducted to identify and analyze other important factors.

## Figures and Tables

**Table 1:** The methodological criteria.

Criterion	Possible Outcome
“Is the research question well stated?”	Yes/No
2. “Is the sample/population identified and appropriate?”	Yes/No
3. “Are the inclusion/exclusion criteria described and appropriate?”	Yes/No or Not applicable
4. “If applicable, is the participation rate reported and appropriate?”	Yes/No or Not applicable
5. “Is the same data collection method used for all respondents?”	Yes/No
6. “Are the variables important, well measured, valid, and reliable?”	Yes/No or Not applicable
7. “Is the outcome defined and measurable?”	Yes/No
8. “Is the statistical analysis appropriate?”	Yes/No or Not applicable

Source: [34,35].

**Table 2:** Basic information of the reviewed articles.

Code Number	Title	Authors	Journal	Publication Year	Number of Citations *
1	The Role of Perceived Environmental Responsibility and Environmental Concern on Shaping Green Purchase Intention	Duong et al. [36]	*Vision*	2022	0
2	Consumer Confidence and Green Purchase Intention: An Application of the Stimulus–Organism–Response Model	Han et al. [37]	*Journal of Retailing and Consumer Services*	2022	1
3	“Why Do We Buy Green Products?” An Extended Theory of the Planned Behavior Model for Green Product Purchase Behavior	Kamalanon et al. [38]	*Sustainability*	2022	3
4	Analysing the Influence of Companies’ Green Communication in College Students’ Green Purchase Behaviour: An Application of the Extended Theory of Planned Behaviour Model	Sousa et al. [39]	*Administrative Sciences*	2022	0
5	Investigating the Impact of Green Marketing Components on Purchase Intention: The Mediating Role of Brand Image and Brand Trust	Tan et al. [40]	*Sustainability*	2022	1
6	Consumer Belief System and Pro-Environmental Purchase Intention: Does Psychological Distance Intervene?	Shabnam et al. [41]	*Journal of Cleaner Production*	2021	1
7	Social Media and Sustainable Purchasing Attitude: Role of Trust in Social Media and Environmental Effectiveness	Zafar et al. [42]	*Journal of Retailing and Consumer Services*	2021	20
8	Green Consumption in Vietnam: Effects of Eco-Certification, Brand, and Moderate Incongruity of their Origins on Purchase Intent	Dekhili and Nguyen [43]	*Recherche et Applications en Marketing (English Edition)*	2021	3
9	Fostering a Clean and Sustainable Environment through Green Product Purchasing Behavior: Insights from Malaysian Consumers’ Perspective	Al-Kumaim, et al. [44]	*Sustainability*	2021	3
10	Green Product Awareness Effect on Green Purchase Intentions of University Students: An Emerging Market’s Perspective	Ansu-Mensah [45]	*Future Business Journal*	2021	2
11	Which are the Determinants of Green Purchase Behaviour? A Study of Italian Consumers	Dangelico, et al. [46]	*Business Strategy and the Environment*	2021	23
12	Greener than Others? Exploring Generational Differences in Green Purchase Intent	Ham, et al. [47]	*International Journal of Market Research*	2021	2
13	Greenwash and Green Purchase Behavior: An Environmentally Sustainable Perspective	Hameed, et al. [48]	*Environment, Development and Sustainability*	2021	23
14	Ecolabels and the Attitude–Behavior Relationship towards Green Product Purchase: A Multiple Mediation Model	Riskos, et al. [49]	*Sustainability*	2021	13
15	Consumers’ purchase behaviour and green marketing: A synthesis, review, and agenda	Sharma [50]	*International Journal of Consumer Studies*	2021	25
16	Impact of Green Trust and Green Perceived Quality on Green Purchase Intentions: A Moderation Study	Wasaya, et al. [51]	*Environment, Development and Sustainability*	2021	14
17	Green Purchase Behavior: The Effectiveness of Sociodemographic Variables for Explaining Green Purchases in Emerging Market	Witek and Kuzniar [52]	*Sustainability*	2021	45
18	Consumers Purchase Intentions of Green Electric Vehicles: The Influence of Consumers Technological and Environmental Considerations	Dutta and Hwang [53]	*Sustainability*	2021	7
19	Green Purchase Intention: Effects of Electronic Service Quality and Customer Green Psychology	Ahmad and Zhang [54]	*Journal of Cleaner Production*	2020	65
20	Why Do Consumers Make Green Purchase Decisions? Insights from a Systematic Review	Zhang and Dong [55]	*International Journal of Environmental Research and Public Health*	2020	77
21	The Influence of Green Brand Affect on Green Purchase Intentions: The Mediation Effects of Green Brand Associations and Green Brand Attitude	Chen, et al. [56]	*International Journal of Environmental Research and Public Health*	2020	26
22	Green Consumption: Strategic Retail Considerations and Consumer Confidence	D’Souza, et al. [57]	*Journal of Strategic Marketing*	2020	1
23	Knowledge Foundation in Green Purchase Behaviour: Multidimensional Scaling Method	Marvi, et al. [58]	*Cogent Business and Management*	2020	9
24	The Effects of Consumer Attitude on Green Purchase Intention: A Meta-Analytic Path Analysis	Zaremohzzabieh, et al. [59]	*Journal of Business Research*	2020	49
25	Impressing My Friends: The Role of Social Value in Green Purchasing Attitude for Youthful Consumers	Caniëls, et al. [60]	*Journal of Cleaner Production*	2020	11
26	Consumer Antecedents Towards Green Product Purchase Intentions	Costa, et al. [61]	*Journal of Cleaner Production*	2020	22
27	Listen to Others or Yourself? The Role of Personal Norms on the Effectiveness of Social Norm Interventions to Change Pro-Environmental Behavior	de Groot, et al. [62]	*Journal of Environmental Psychology*	2020	9
28	Residents’ Green Purchasing Intentions in a Developing-Country Context: Integrating PLS-SEM and MGA Methods	Wang, et al. [63]	*Sustainability*	2019	26
29	An extended Model of Value–Attitude–Behavior to Explain Chinese Consumers’ Green Purchase Behavior	Cheung and To [64]	*Journal of Retailing and Consumer Services*	2019	180
30	Exploring Green Purchasing Behaviour among College Students in a Developing Economy	Ndofirepi and Matema [65]	*Southern African Business Review*	2019	3
31	How Does Green Product Knowledge Effectively Promote Green Purchase Intention?	Wang, et al. [66]	*Sustainability*	2019	65
32	Exploring the Consumer Behavior of Intention toPurchase Green Products in Belt and Road Countries: An Empirical Analysis	Chen, et al. [5]	*Sustainability*	2018	139
33	Impact of Religious Values and Habit on an Extended Green Purchase Behaviour Model	Ghazali, et al. [67]	*International Journal of Consumer Studies*	2018	43
34	Trust to Go Green: An Exploration of Consumer Intentions for Eco-friendly Convenience Food	Ricci, et al. [68]	*Ecological Economics*	2018	133
35	Predictors of Purchase Intention toward Green Apparel Products: A Cross-Cultural Investigation in the US and China	Ko, et al. [69]	*Journal of Fashion Marketing and Management: An 36 International Journal*	2017	146
36	Determinants of Consumers’ Purchase Behaviour towards Green Brands	Wang [70]	*The Service Industries Journal*	2017	47
37	Impact of Environmental Knowledge and Product Quality on Student Attitude toward Products with Recycled/Remanufactured Content: Implications for Environmental Education and Green Manufacturing	Sun, et al. [71]	*Business Strategy and the Environment*	2017	62

Note: * total number of citations on Google Scholar on 1 August 2022.

## Data Availability

Not applicable.
